# Systematics and Molecular Phylogeny of the Family Oscarellidae (Homoscleromorpha) with Description of Two New *Oscarella* Species

**DOI:** 10.1371/journal.pone.0063976

**Published:** 2013-05-30

**Authors:** Eve Gazave, Dennis V. Lavrov, Jory Cabrol, Emmanuelle Renard, Caroline Rocher, Jean Vacelet, Maja Adamska, Carole Borchiellini, Alexander V. Ereskovsky

**Affiliations:** 1 Institut Jacques Monod, CNRS, UMR 7592, Université Paris Diderot, Sorbonne Paris Cité, Paris, France; 2 Aix-Marseille Université, CNRS, UMR 7263, Mediterranean Institute of Biodiversity and Ecology (IMBE), Marseille, France; 3 Department of Ecology, Evolution, and Organismal Biology, Iowa State University, Ames, Iowa, United States of America; 4 Sars International Centre for Marine Molecular Biology, Bergen, Norway; 5 Department of Embryology, Faculty of Biology and Soil Science, St-Petersburg State University, Saint-Petersburg, Russia; Australian Museum, Australia

## Abstract

The family Oscarellidae is one of the two families in the class Homoscleromorpha (phylum Porifera) and is characterized by the absence of a skeleton and the presence of a specific mitochondrial gene, *tatC*. This family currently encompasses sponges in two genera: *Oscarella* with 17 described species and *Pseudocorticium* with one described species. Although sponges in this group are relatively well-studied, phylogenetic relationships among members of Oscarellidae and the validity of genus *Pseudocorticium* remain open questions. Here we present a phylogenetic analysis of Oscarellidae using four markers (18S rDNA, 28S rDNA, *atp6*, *tatC*), and argue that it should become a mono-generic family, with *Pseudocorticium* being synonymized with *Oscarella*, and with the transfer of *Pseudocorticium jarrei* to *Oscarella jarrei*. We show that the genus *Oscarella* can be subdivided into four clades, each of which is supported by either a small number of morphological characters or by molecular synapomorphies. In addition, we describe two new species of *Oscarella* from Norwegian fjords: *O. bergenensis* sp. nov. and *O. nicolae* sp. nov., and we compare their morphology, anatomy, and cytology with other species in this genus. Internal anatomical characters are similar in both species, but details of external morphology and particularly of cytological characters provide diagnostic features. Our study also confirms that *O. lobularis* and *O. tuberculata* are two distinct polychromic sibling species. This study highlights the difficulties of species identification in skeleton-less sponges and, more generally, in groups where morphological characters are scarce. Adopting a multi-marker approach is thus highly suitable for these groups.

## Introduction

Sponges (phylum Porifera) are now formally regarded as being composed of four lineages: Demospongiae, Calcarea, Hexactinellida and Homoscleromorpha [1], [2], [3], [4], [5]. The latter group, Homoscleromorpha, contains about 78 species and is subdivided into two families: Plakinidae and Oscarellidae [1], [6]. Currently, family Plakinidae encompasses five spiculate genera (*Plakina, Plakortis, Plakinastrella, Placinolopha* and *Corticium*) while Oscarellidae includes two aspiculate genera (*Oscarella* and *Pseudocorticium*) [1], [6]. Family Oscarellidae [6] was established by Lendenfeld in 1887, but was rejected in 1995 [7] following the description of *Pseudocorticium*, which is morphologically similar to *Corticium* though lacking spicules. Oscarellidae was restored as a family only recently as the result of studies of molecular phylogeny [6] and metabolomic fingerprints [8] of Homoscleromorpha. The results of these studies have demonstrated that morphological similarities found in spiculate *Corticium* and aspiculate *Pseudocorticium* (cortex, aquiferous system organization, and outer morphology) are either plesiomorphic or homoplasic characters [6] and that *Pseudocorticium* is, in fact, more closely related to *Oscarella* species with very different internal and external morphology.

Although the subdivision of Homoscleromorpha into two families is now formally accepted by the sponge scientific community [1], [6], [9], the relationships between the two oscarellid genera and consequently, the monophyly of *Oscarella,* remain contentious. Indeed, in our previous study [6], the analyses of complete mtDNA genomes and 18S rDNA data supported the paraphyly of the *Oscarella,* which encompassed *Pseudocorticium jarrei* Boury-Esnault et al., 1995. In contrast, the 28S rDNA sequences supported the monophyly of *Oscarella* with *P. jarrei* as its sister group. In order to resolve this issue, we conducted a further molecular study including additional *Oscarella* species.

*Oscarella lobularis* (Schmidt, 1862) [10], the type species of the genus, was long considered to be a single abundant cosmopolitan species displaying a high polymorphism of both consistency (soft and cartilaginous) and color (purple, blue, yellow and green) [11]. In 1992, Boury-Esnault and colleagues investigated the relationships among four color morphotypes of *O. lobularis* from the Marseille area by analyzing their allozymes and cytological features [12]. They showed that two species were present rather than one: *O. lobularis* (the soft purple/ivory specimens, [13]) and *O. tuberculata* (Schmidt, 1868) (the yellow, green or blue cartilaginous specimens). Since then, however, the picture has become more complex and is rife with ambiguities. Recent studies have found soft specimens of *Oscarella* which do not have the habitual purple/ivory coloring, but which are blue, entirely purple or pink. Similarly, cartilaginous specimens of *Oscarella* may also be purple or pink in addition to the green, blue and yellow morphotypes [13], [14]. Subsequent, finer histological studies revealed additional differences among various color morphs of *Oscarella,* and the ‘cosmopolitan’ *O. lobularis* turns out to be different species (10 new species of *Oscarella* have been described during the last 20 years) [15], [16], [17], [18]. The absence of a skeleton (the main morphological character for sponge taxonomy) and thus, the paucity of available morphological characters for *Oscarella* systematics, is largely responsible for difficulties associated with species delimitation in this genus, as well as in other genera of sponges without skeletons (e.g. *Halisarca* [19], [20], [21]). At present, *Oscarella* comprises 17 species, listed in the World Porifera Data Base (http://www.marinespecies.org/porifera/index.php), including seven Mediterranean species. However, this is certainly an underestimate and several new species are currently under description (this study) or have yet to be described. The relationships among these 17 *Oscarella* species and the phylogenetic position of *Pseudocorticium jarrei* relative to them are also largely unknown [6]. In addition, the relationships between the different color morphs of the two putative sibling species *O. lobularis* and *O. tuberculata* [14] have not yet been fully resolved and more loci from more color morphs are needed to elucidate them. This is crucially important, especially because *O. lobularis* is being developed as a new model species for evo-devo studies [13], [22], [23], [24], [25].

Thus the aim of this paper is to investigate the principal uncertainties in the phylogeny of Oscarellidae described above: (i) the position of *Pseudocorticium jarrei* and the monophyly of genus *Oscarella*, (ii) the relationships among common *Oscarella* species, (iii) the relationships between different color morphs of *Oscarella lobularis* and *O. tuberculata*. For this purpose, we collected a diverse dataset relating to 22 Oscarellidae specimens from different geographical areas. Our dataset includes four molecular markers (two nuclear (18S rDNA, 28S rDNA) and two mitochondrial (*atp6*, *tatC)*), predicted secondary structures features for nuclear rDNAs [26] and multiple non-molecular characters (in particular, histological and cytological). We also incorporated two new species of *Oscarella* from Bergen Fjords in our analysis, and we provide their morphological descriptions and formal diagnoses. We discuss our results from an integrative taxonomic point of view [27].

## Methods

### 1. Specimen Collection

**Figure 1:**
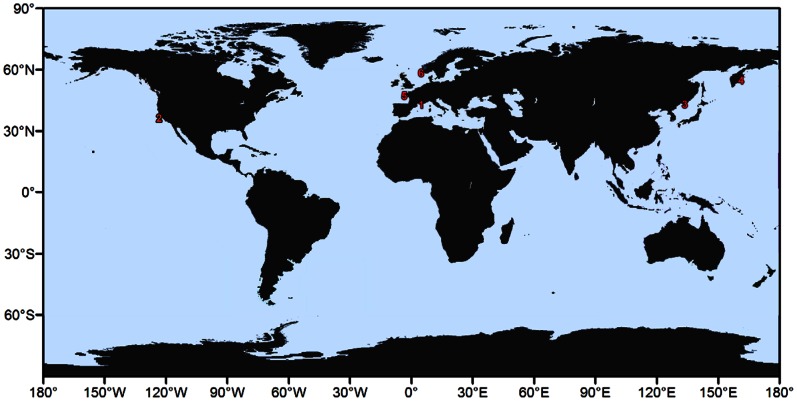
Map of the collection sites for this study. The numbers refer to the species locations detailed in Table 1.

Specimens of Oscarellidae from the Mediterranean Sea, the Norwegian Fjords, the East Atlantic and the North Pacific were collected using SCUBA diving by members of our team (AVE, EG) or were provided by colleagues (see Acknowledgments). Locations of the collection sites are shown on Figure 1. The samples used in this study and their current taxonomic status are summarized in Table 1. The different color morphs of *Oscarella lobularis* and *O. tuberculata* are presented *in situ* in Figure S1. We obtained molecular data from 9 of the 17 officially described species of *Oscarella*. In addition, we formally describe two new species in this paper and discuss two specimens with uncertain systematic positions (*Oscarella* sp. (pink) and *Oscarella* sp. (purple)). Despite substantial efforts, we did not succeed in obtaining DNA from museum specimens of *O. nigraviolacea* Bergquist & Kelly, 2004, *O. ochreacea* Muricy & Pearse, 2004 and *O. stillans* Bergquist & Kelly, 2004; likely due to problems with DNA preservation [28], [29]. We choose not to include in our sampling *O. imperialis* Muricy et al., 1996 [30], as we were unable to find this species with certainty *in situ*.

**Table 1:** List of species/specimens used in this work according to the classification of Systema Porifera [89] and the recent updates added to the World Porifera Database [9].

		GenBank Accession numbers					Collection sites	
Species/color morphs		18S rDNA	28S rDNA	*atp6*	*tatC*	Complete mt	Names	Map n°
***Oscarella***								
*Oscarella lobularis* purple/ivory	[10]	HM118536	HM118549	HQ269361	HQ269361	HQ269361	Marseille, France(Coral cave)	1
*Oscarella lobularis* purple	[10]	**JX462755**	**JX462774**	**JX975205**	**JX975192**	–	Marseille, France(Passe Tiboulen)	1
*Oscarella lobularis* pink	[10]	**JX462757**	**JX462776**	**JX975204**	**JX975191**	–	Marseille, France(Passe Tiboulen)	1
*Oscarella lobularis* blue	[10]	**JX462756**	**JX462775**	**JX975203**	**JX975190**	–	Marseille, France(Passe Tiboulen)	1
*Oscarella tuberculata* yellow	[88]	–	–	**JX963639**	**JX963639**	**JX963639**	Marseille, France(Passe Tiboulen)	1
*Oscarella tuberculata* green	[88]	**JX462761**	**JX462777**	HQ269353	HQ269353	HQ269353	Marseille, France(La Vesse)	1
*Oscarella tuberculata* pink	[88]	**JX462759**	**JX462779**	**JX975207**	**JX975194**	–	Marseille, France(La Vesse)	1
*Oscarella tuberculata* purple	[88]	**JX462758**	**JX462780**	**JX975208**	**JX975195**	–	Marseille, France(La Vesse)	1
*Oscarella tuberculata* blue	[88]	**JX462760**	**JX462778**	–	**JX975193**	–	Marseille, France(La Vesse)	1
*Oscarella carmela*	[29]	EU702422	EF654519	NC_009090	NC_009090	NC_009090	California, USA(Carmel Bay)	2
*Oscarella malakhovi*	[15]	HM118537	HM118550	HQ269364	HQ269364	HQ269364	Japan Sea, Russia(Vostok Bay)	3
*Oscarella viridis*	[30]	**JX462764**	–	HQ269358	HQ269358	HQ269358	Marseille, France(Jarre Cave)	1
*Oscarella microlobata*	[30]	HM118538	HM118551	HQ269355	HQ269355	HQ269355	Marseille, France(Jarre Cave)	1
*Oscarella kamchatkensis*	[16]	**JX462762**	**JX462781**	**JX975202**	**JX975189**	–	Avacha Gulf,Kamchatka(Starichkov Island)	4
*Oscarella* sp. (purple)	n/a	**JX462766**	**JX462782**	**JX963639**	**JX963639**	**JX963640**	Marseille, France(Maire Island)	1
*Oscarella rubra*	[58]	**JX462765**	**JX462773**	**JX975206**	**JX975197**	–	Ria d’Etel, France	5
*Oscarella balibaloi*	[17]	**JX462763**	**-**	**JX975198**	**JX975196**	–	Marseille, France(Coral Cave)	1
*Oscarella nicolae* sp. nov.	–	**JX462769**	**JX462770**	**JX975199**	**JX975186**	–	Bergen, Norway(Skarvoysundet)	6
*Oscarella* sp. (pink)	n/a	**JX462767**	**JX462772**	**JX975200**	**JX975187**	–	Bergen, Norway(Skarvoysundet)	6
*Oscarella bergenensis*sp. nov.	–	**JX462768**	**JX462771**	**JX975201**	**JX975188**	–	Bergen, Norway(Skarvoysundet)	6
***Pseudocorticium***								
*Pseudocorticium jarrei*	[7]	HM118539	HM118552	HQ269357	HQ269357	HQ269357	Marseille, France(Jarre Cave)	1

The collection sites and the GenBank accession numbers of the four markers and of the complete mitochondrial genomes are indicated. In the sequence column, the new sequence accession numbers are written in bold.

### 2. Morphological Studies

#### 2.1. Taxonomy

The identification of all specimens has been carefully checked on the basis of morphological characters by the taxonomists in our team (AVE and JV).

#### 2.2. Description of new species

Specimens from Norway were collected using SCUBA diving on June 23 2009 from vertical walls of granite rocks (North Sea, Norway, Skarvoysundet +60° 27' 34.74" N, +4° 56' 2.16" E) at depths of 3 to 9 m. Vouchers for transmission electron microscopy (TEM) and scanning electron microscopy (SEM) were fixed according to [31]. Sections were cut with a diamond knife on a Leica Ultracut UCT Ultramicrotome. Semi-thin sections were stained with toluidine blue, observed using light microscopy (LM) and photographed with a Leica DMLB digital camera. For SEM, the specimens were fractured in liquid nitrogen, critical-point-dried, sputter-coated with gold-palladium, and observed under a Hitachi S570 SEM. Type specimens have been deposited in the Muséum National d'Histoire Naturelle (MNHN, Paris, France) and the new species have been registered in ZooBank.

#### 2.3. Nomenclatural Acts

The electronic edition of this article conforms to the requirements of the amended International Code of Zoological Nomenclature, and hence the new names contained herein are available under that Code from the electronic edition of this article. This published work and the nomenclatural acts it contains have been registered in ZooBank, the online registration system for the ICZN. The ZooBank LSIDs (Life Science Identifiers) can be resolved and the associated information viewed through any standard web browser by appending the LSID to the prefix "http://zoobank.org/". The LSID for this publication is: urn:lsid:zoobank.org:pub: **9766D4A6-D2B5-4311-BFDB-8B3262B401F1**. The electronic edition of this work was published in a journal with an ISSN, and has been archived and is available from the following digital repositories: PubMed Central and LOCKSS.

#### 2.4. Non-molecular characters

To identify putative non-molecular synapomorphies for clades revealed by molecular analyses, several characters belonging to five major categories - (i) ecology/geography, (ii) external morphology, (iii) histology/cytology, (iv) microbiology and (v) embryology - are described for each species. Observed characters and their states are listed in Tables 2 and 3. Most of these characters are non-informative for phylogenetic reconstruction and thus this table of characters has not been used as a matrix for phylogenetic analyses. Instead, parsimony reconstruction of character evolution (the matrix of characters is provided in Text S1 Text S1) based on the consensus molecular tree (Figure S2) was performed using Mesquite software version 2.72 [32].

**Table 2:** Three sets of non-molecular characters-states for each species/specimen: ecology/geography, external morphology and associated microbes.

Clade		Ecology/Geography		External morphology			Associated microbes
		Locality	Habitat	Color	Consistency	Surface	Density ofbacteria
**A**	***O. balibaloi***	Med	Semi-obscurecaves	White Orange	Soft mucous Slimy	Corrugated Lumpy microlobate	LMA
**A**	***O. kamchatkensis***	N-W Pacific	Boulders Rocks	Orange Yellow	Soft slimy	Lumpy Microlobate	LMA
**A**	***O. nicolae*** ** sp. nov.**	E North Sea	Rocks Algae	Ivory yellowish	Delicate mucous	Microlobate	LMA
**A**	***Pseudocorticium jarrei***	Med	Obscure caves	Cream	Firm Cartilaginous	Smooth Slippery Corrugate Folded	HMA
**B**	***O. viridis***	Med	Obscure caves	Light green	Soft fragile	Rugose	LMA
**C**	***O. malakhovi***	N-W Pacific	Bivalve shells Stones	Pinky Yellow	Soft slimy	Lumpy Undulated Microlobate	LMA
**C**	***O. carmela***	N-E Pacific	Boulders	Light brown Orange	Soft slimy	Lumpy Microlobate	LMA
**D**	***O. lobularis***	Med	Semi-obscurecaves, Walls	Variable	Soft	Smooth	LMA
**D**	***O. tuberculata***	Med	Semi-obscurecaves Walls	Variable	Cartilaginous	Wrinkled	LMA
**D**	***O. rubra***	E Atlantic	Bivalve shells Stones	Yellow Red Orange	Soft cartilaginous	Lumpy Microlobate	LMA
**D**	***O.*** ** sp. (purple)**	Med	Bryozoan sand bottom	Purple	Soft	Microlobate	LMA
**D**	***O.*** ** sp. (pink)**	E North Sea	Rocks Algae	Pink	Delicate Resilience	Smooth Small wrinkles	LMA
**D**	***O. bergenensis*** ** sp. nov.**	E North Sea	Rocks Algae	Red Orange	Soft	Smooth Small wrinkles	LMA
**?**	***O. microlobata***	Med	Obscure caves	Light brown	Soft fragile	Rugose	HMA

Clades defined by molecular data are indicated (a “?” is given for *O. microlobata* for which the position is unclear). Med: Mediterranean Sea; LMA: low microbial abundance; HMA: high microbial abundance.

**Table 3:** Histology, cytology and embryology morphological characters-states for each species/specimen.

Clade		Histology/Cytology									Embryology		
		Cortex	Canalsystem	Choanocytechambers	Archaeocyte	Vacuolarcells	Granularcells	Spherulouscells	Spherulouscells withpara-crystallineinclusions	Basementmembrane	Cinctoblastulalarva	Multipolaringression	Asynchronousspermatogenesis
**A**	***O. balibaloi***	No	Sylleibid	Eurypylous	No	1T	1T	No	1T	Yes	Yes	Yes	Yes
**A**	***O. kamchatkensis***	No	Sylleibid	Eurypylous	No	No	2T	No	1T	Yes	Yes	Yes	Yes
**A**	***O. nicolae sp. nov.***	No	Sylleibid	Eurypylous	Yes	No	1T	No	1T	Yes	Yes	Yes	Yes
**A**	***Pseudocorticium*** ***jarrei***	Yes	Leuconoid	Diplodal	No	No	3T	No	1T	Yes	Yes	Yes	Yes
**B**	***O. viridis***	No	Sylleibid	Eurypylous	Yes	1T	1T	No	No	Yes	Yes	Yes	Yes
**C**	***O. malakhovi***	No	Sylleibid	Eurypylous	Rare	1T	1T	No	No	Yes	Yes	Yes	Yes
**C**	***O. carmela***	No	Sylleibid	Eurypylous	Yes	1T	1T	No	No	Yes	Yes	Yes	Yes
**D**	***O. lobularis***	No	Sylleibid	Eurypylous	No	2T	No	No	No	Yes	Yes	Yes	Yes
**D**	***O. tuberculata***	No	Sylleibid	Eurypylous	Yes	1T	No	No	No	Yes	Yes	Yes	Yes
**D**	***O. rubra***	No	Sylleibid	Eurypylous	No	1T	1T	No	No	Yes	Yes	?	Yes
**D**	***O. sp. (purple)***	No	Sylleibid	Eurypylous	Yes	1T	1T	No	No	Yes	Yes	?	?
**D**	***O. sp. (pink)***	No	Sylleibid	Eurypylous	Rare	1T	1T	No	No	Yes	Yes	?	?
**D**	***O. bergenensis*** ***sp. nov.***	No	Sylleibid	Eurypylous	Rare	1T	1T	No	No	Yes	Yes	?	?
**−**	***O. microlobata***	No	Sylleibid	Eurypylous	No	1T	1T	1T	1T	Yes	Yes	Yes	Yes

Clades defined by molecular data are indicated. 1T: one type; 2T: two types: 3T: three types.

### 3. Molecular Methods

#### 3.1. Rationale for the choice of molecular markers

Our decision to use 18S rDNA and 28S rDNA markers in our analysis was based on their prior efficacy in solving phylogenetic relationships at the genus and supra-generic levels for various sponge groups [2], [6], [33], [34], [35], [36], [37]. In addition, we developed two new mitochondrial markers for Homoscleromorpha based on our previous data [6] and on new complete mtDNA sequences from *Oscarella tuberculata* yellow and *Oscarella* sp. (purple). The regions chosen for these markers are located within *tatC* and *atp6* and contain the largest number of parsimony informative sites per kb of sequence in a whole mt-genome alignment for five closely-related *Oscarella* species: *O. tuberculata* green, *O. tuberculata* yellow, *Oscarella* sp. (purple), *O. lobularis*, and *O. viridis.* Together, these two markers encompassed ∼30% of such sites (10/35) in fewer than 8% of mt-genome sequences.

Interestingly, *atp6* has recently been used in another study where it has been shown to be suitable for alpha-level systematics in sponges [38]. To circumvent the pitfalls of using single-gene trees, which can tell a biased story of the species relationships [38], [39], we combined these four genes in our analysis.

#### 3.2. DNA sequence acquisition

Procedures used for genomic DNA extraction, cloning and DNA sequencing are standard laboratory protocols described in previous publications [6], [37]. PCR primers for full-length/partial 18S rDNA, partial 28S rDNA, *atp6* and *tatC* amplification are provided in Table S1. It was necessary to adapt reaction conditions for each species from previous studies [6], [37]; the exact conditions of amplification can be provided by the authors upon request. The poriferan origin of the sequences was checked by a BLAST search [40] against the NCBI GenBank collection (http://www.ncbi.nlm.nih.gov/). All new sequences/genomes were deposited in GenBank under accession numbers listed in Table 1.

#### 3.3. Sequence alignment

To achieve a reasonable trade-off between representativeness of outgroup taxa and ease of alignment, and because our prime interests were relationships within the Oscarellidae, we restricted our sampling to Homoscleromorpha. Several species of Plakinidae (three *Corticium* and two *Plakortis* species), the sister group of Oscarellidae, were used as an outgroup. Initial sequence alignment was performed using the software MUSCLE online (http://www.ebi.ac.uk/Tools/muscle/index.html) [41], [42], and was subsequently optimized by eye using the Bioedit Sequence Alignment Editor v5.09 [43]. Ambiguously aligned regions were determined by Gblocks v0.91 b software [44] for nuclear markers only (mitochondrial ones were partitioned by codon position). A relaxed selection of blocks is better for short alignment [45], thus the settings were the following for the 18S rDNA [1∶13; 2∶13; 3∶8 4∶2; 5: all] and the 28S rDNA [1∶13; 2∶13; 3∶8; 4∶2; 5: all]. The treatment by Gblocks resulted in the removal of 1% and 4%, for the 18S rDNA and 28S rDNA alignments, respectively. The character exclusion sets based on Gblocks are available upon request from the corresponding author.

#### 3.4. Phylogenetic analyses

Phylogenetic analyses were performed using maximum likelihood (ML) and Bayesian inference (BI) methods.

For ML analyses, we used the Akaike Information Criterion (AIC) in JModelTest [46] to determine the best fitting nucleotide substitution model for each data set. The following models were chosen for 18S rDNA, 28S rDNA, 18S rDNA +28S rDNA, *atp6*, *tatC*, *tatC*+*atp6*, 18S rDNA +28S rDNA +*tatC*+*atp6* datasets respectively: TIM2+G; GTR+G; TIM2+G; TPM2uf+I+G; TIM1+I; GTR+G; GTR+G. ML phylogenetic analyses were performed with PhyML software v.3 [47], [48] using the previously estimated parameters. Among sites, rate heterogeneity was estimated using a discrete approximation of the gamma distribution with six rate categories. Gaps were treated as missing data and the statistical robustness of the tree topology was assessed by non-parametric bootstrap resampling (1000 replicates) [49]. Bootstrap values >80 were considered high enough to support clades in ML reconstructions.

BI analyses were performed with MrBayes 3.2.1 [50] under the best-fit evolutionary model estimated for each independent gene or partition under the AIC criterion with MrModeltest 2.3 [51]. The models selected for 18S rDNA and 28S rDNA were GTR+I+G and GTR+G, respectively. For mitochondrial markers *atp6* and *tatC*, we partitioned the dataset according to codon position and the models selected are as follows; [*atp6*_1^st^ position: GTR+I, *atp6*_2^nd^ position: GTR+I, *atp6*_3^rd^ position: GTR+G]; [*tatC*_1^st^ position: GTR+I, *tatC*_2^nd^ position: GTR+I, *tatC*_3^rd^ position: HKY+I].

Four Markov Chains were run for 2 million generations and sampled every 100 generations. The chains converged significantly and the average standard deviation of split frequencies was <0.01 at the end of the run. The trees of the early generations (5000 trees) were discarded until the probabilities reached a stable plateau (burn-in) and the remaining trees were used to generate a 50% majority-rule consensus tree. Only posterior probabilities >0.90 were considered to robustly support clades.

All trees except the *tatC* and *tatC*+*atp6* were rooted on Plakinidae species, the exceptions arising as the *tatC* gene is specific to Oscarellidae [6]. Based on the nuclear and *atp6* phylogenetic analyses (this study) and previous complete mitochondrial analyses [6], these trees were instead rooted on a robust internal clade. The trees were visualized and edited using FigTree v.1.3.1 [52].

#### 3.5. Secondary structure analysis of 18S rDNA sequences and their optimization on the 18S rDNA molecular tree

The MFold server (http://mobyle.pasteur.fr/cgi-bin/MobylePortal/portal.py?form=mfold [53] was used to determine secondary structures of V4 variable regions of the 18S rDNA for all species. This region was defined as covering helix 43 [54], [55]. The default settings for all parameters were used as in a previous study [37]. When multiple secondary structures were found, we chose the structure with the lowest free energy (ΔG in kcal/mol) [26].We decomposed the rDNA secondary structures into elements (framed by different colors) and defined binary characters (*i.e.* presence/absence of each element). A matrix (Text S2) was constructed and parsimony reconstructions of character evolution were performed using Mesquite software version 2.72 [32] on the 18S rDNA molecular tree.

#### 3.6. Sequences identity and nucleotide diversities

We investigated the percentage of molecular divergence (Table 4) on the mitochondrial marker sequences (*atp6* and *tatC*) by using the identity matrix option of the Bioedit software [43]. Nucleotide diversities (π) between *Oscarella tuberculata*, *O. lobularis*, D3 sequences, as well as congeneric species of *Oscarella,* were estimated using DnaSP 5.10 software [56] for the *atp6* marker and compared to available demosponge data [38] (Table 5).

**Table 4:** Identity values between members of clade D for mitochondrial markers.

	*O. tub*_ blue	*O. tub*_ green	*O. tub*_ yellow	*O. tub*_ pink	*O. tub*_ purple	*O. rubra*	*O*. sp. (purple)	*O*. sp.(pink)	*O. bergen-ensis*	*O. lob*_ purple/ivory	*O. lob*_ pink	*O. lob*_ purple	*O. lob*_ blue
***O. tub*** **_ blue**	ID												
***O. tub*** **_ green**	−/0.998	ID											
***O. tub*** **_ yellow**	−/0.994	0.996/0.995	ID										
***O. tub*** **_ pink**	−/0.998	1/1	0.996/0.995	ID									
***O. tub*** **_ purple**	−/0.998	1/1	0.996/0.995	1/1	ID								
***O. rubra***	−/0.989	0.996/0.991	0.996/0.992	0.996/0.991	0.996/0.991	ID							
***O*** **. sp. (purple)**	−/0.989	0.996/0.991	0.996/0.992	0.996/0.991	0.996/0.991	1/1	ID						
***O*** **. sp. (pink)**	−/0.989	0.996/0.991	0.996/0.992	0.996/0.991	0.996/0.991	1/1	1/1	ID					
***O. bergenensis***	−/0.980	0.990/0.982	0.990/0.983	0.990/0.982	0.990/0.982	0.993/0.985	0.993/0.985	0.993/0.985	ID				
***O. lob*** **_** purple//ivory	−/0.986	0.992/0.988	0.992/0.989	0.992/0.988	0.992/0.988	0.995/0.991	0.995/0.991	0.995/0.991	0.995/0.982	ID			
***O. lob*** **_ pink**	−/0.989	0.992/0.991	0.992/0.992	0.992/0.991	0.992/0.991	0.995/0.994	0.995/0.994	0.995/0.994	0.995/0.985	1/0.994	ID		
***O. lob*** **_ purple**	−/0.991	0.992/0.992	0.992/0.994	0.992/0.992	0.992/0.992	0.995/0.995	0.995/0.995	0.995/0.995	0.995/0.986	1/0.995	1/0.998	ID	
***O. lob*** **_ blue**	−/0.991	0.992/0.992	0.992/0.994	0.992/0.992	0.992/0.992	0.995/0.995	0.995/0.995	0.995/0.995	0.995/0.986	1/0.995	1/0.998	1/1	ID

The upper figure in each cell is for *atp6* and the lower for *tatC*,. *O. lob*: *Oscarella lobularis*; *O. tub*: *O. tuberculata.*

**Table 5:** Nucleotide diversity (π) of *Oscarella lobularis*, *O. tuberculata* and D3 members compared to Demospongiae species for the *atp6* marker.

Species	Number of sequences	Number of populations/localities	π	References
*Amphimedon erina*	3	1	0.000	[38]
*Chondrosia reniformis*	2	1	0.000	[38]
*Cinachyrella* sp.	9	3	0.017	[38]
*Ciona delitrix*	10	2	0.001	[38]
*Placospongia aff. carinata*	14	4	0.001	[38]
*Placospongia aff. melobesioides*	8	2	0.001	[38]
*Oscarella lobularis*	4	1	0.000	This study
*Oscarella tuberculata*	4	1	0.001	This study
D3 sequences	3	−	0.000	This study
*O. lobularis + O. tuberculata*	8	2	0.005	This study

For each species (or cluster), the number of sequences as well as of populations/localities is indicated.

#### 3.7. Consensus tree and diagnostic characters

A consensus tree based on *tatC*+*atp6* molecular phylogenies was manually drawn to map the diverse molecular and non-molecular characters that are diagnostic of some *Oscarella* clades. All robust nodes (BP>50+ PP>0.5) were conserved. Polytomy was prioritized for weakly supported nodes (BP<50 or PP<0.5).

## Results

### 1. Molecular Phylogenies and Analyses

#### 1.1. Phylogenetic analyses of nuclear markers: 18S rDNA and 28S rDNA genes

**Figure 2:**
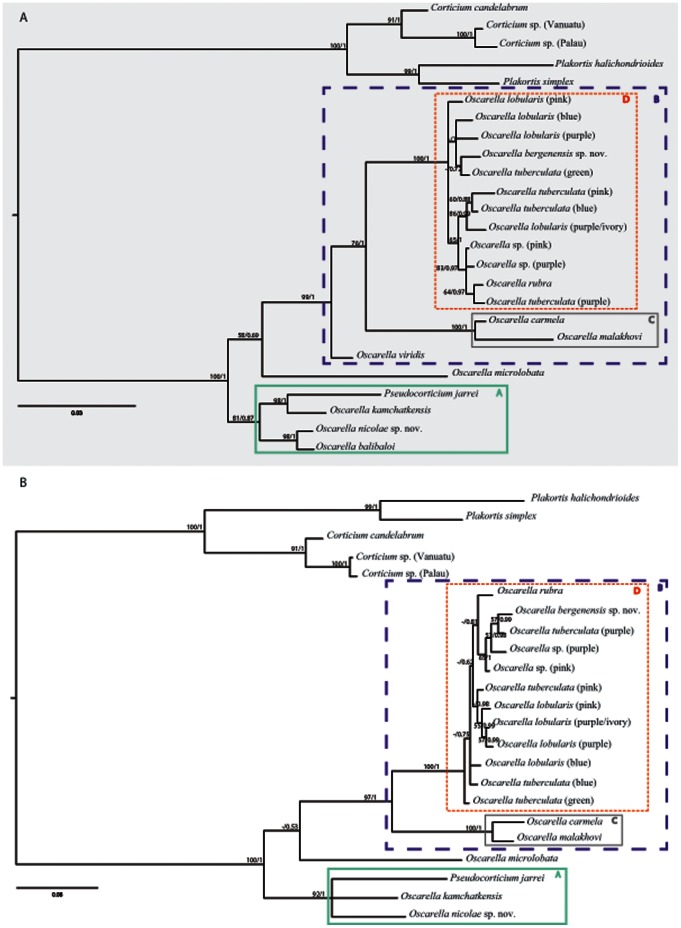
Phylogenetic analysis of nuclear markers. (A) 18S rDNA and (B) 28S rDNA. The topologies presented are posterior consensus trees obtained by the BI analysis using MrBayes. Similar topologies were obtained in ML analysis with PhyML. The numbers are posterior probabilities for BI and bootstrap values (>50) for ML.

The results of the analyses of 18S rDNA and 28S rDNA genes, both separate and combined are mostly congruent and are presented in Figures 2. In the following text section, the bootstraps and posterior probabilities values corresponding to a given node will be listed (in brackets) in the following order: 18S rDNA BP/PP, 28S rDNA BP/PP, 18S rDNA +28S rDNA BP/PP, when relevant. Two well-supported clades named A (81/0.87, 92/1, 96/1) and B (99/1, 97/1, 99/1) are found in these topologies.

Clade A contains *Oscarella balibaloi* Perez et al., 2011, *O. kamchatkensis* Ereskovsky et al., 2009 and *O. nicolae* sp. nov. (see description hereafter)+*Pseudocorticium jarrei*. Although we failed to obtain *O. balibaloi*’s 28S rDNA sequence, 18S rDNA and 18S rDNA +28S rDNA datasets highly support the pairs [*Oscarella nicolae* sp. nov.+*O. balibaloi*] and [*P. jarrei*+*O. kamchatkensis*] as having a sister-group relationship (98/1, 99/1 and 98/1, 83/1). Clade B contains all the other *Oscarella* species except for *Oscarella microlobata* Muricy et al., 1996 which is positioned as the sister group of B but with low support (58/0.69, −/0.53, 88/0.98).

Within clade B we recognize two well-supported clades named C (100/1, 100/1, 100/1) and D (100/1, 100/1, 100/1). Clade C contains *O. carmela* Muricy & Pearce, 2004 and *O. malakhovi* Ereskovsky, 2006. Clade D contains all color morphs of *O. lobularis* and *O. tuberculata*, plus two samples from Bergen (*Oscarella bergenensis* sp. nov. (see description hereafter) and *Oscarella* sp. (pink)), a specimen from the East Atlantic (*O. rubra* (Hanitsch, 1890)) and a sample from the Mediterranean Sea (*Oscarella* sp. (purple)). Both nuclear markers failed to resolve clearly the relationships inside clade D (weak to moderate support in ML, and differences of topology between the three analyses).

*Oscarella viridis* Muricy et al., 1996 (for which only the 18S rDNA sequence was obtained) was placed as the sister group of clade [C+D] (76/1, 97/1, 84/0.99).

In summary, analyses of nuclear markers allow to clearly identify two main clades A and B, the latter being itself composed of two monophyletic groups: C and D. The relationships among the D clade (containing most of our samples) are not always clear and congruent, thus calling for the use of other markers (or more taxa).

#### 1.2. Phylogenetic analyses of mitochondrial markers: tatC and atp6 genes

**Figure 3:**
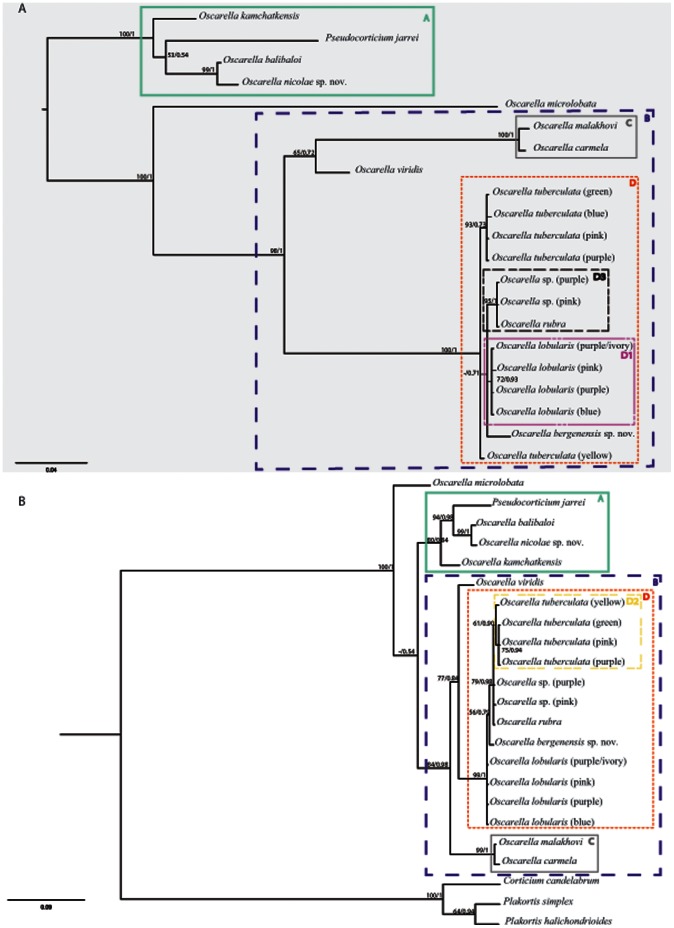
Phylogenetic analysis of mitochondrial markers. (A) *atp6* and (B) *tatC.* The topologies presented are posterior consensus trees obtained by the BI analysis using MrBayes. Similar topologies were obtained in ML analysis with PhyML. The numbers are posterior probabilities for BI and bootstrap values (>50) for ML.

**Figure 4:**
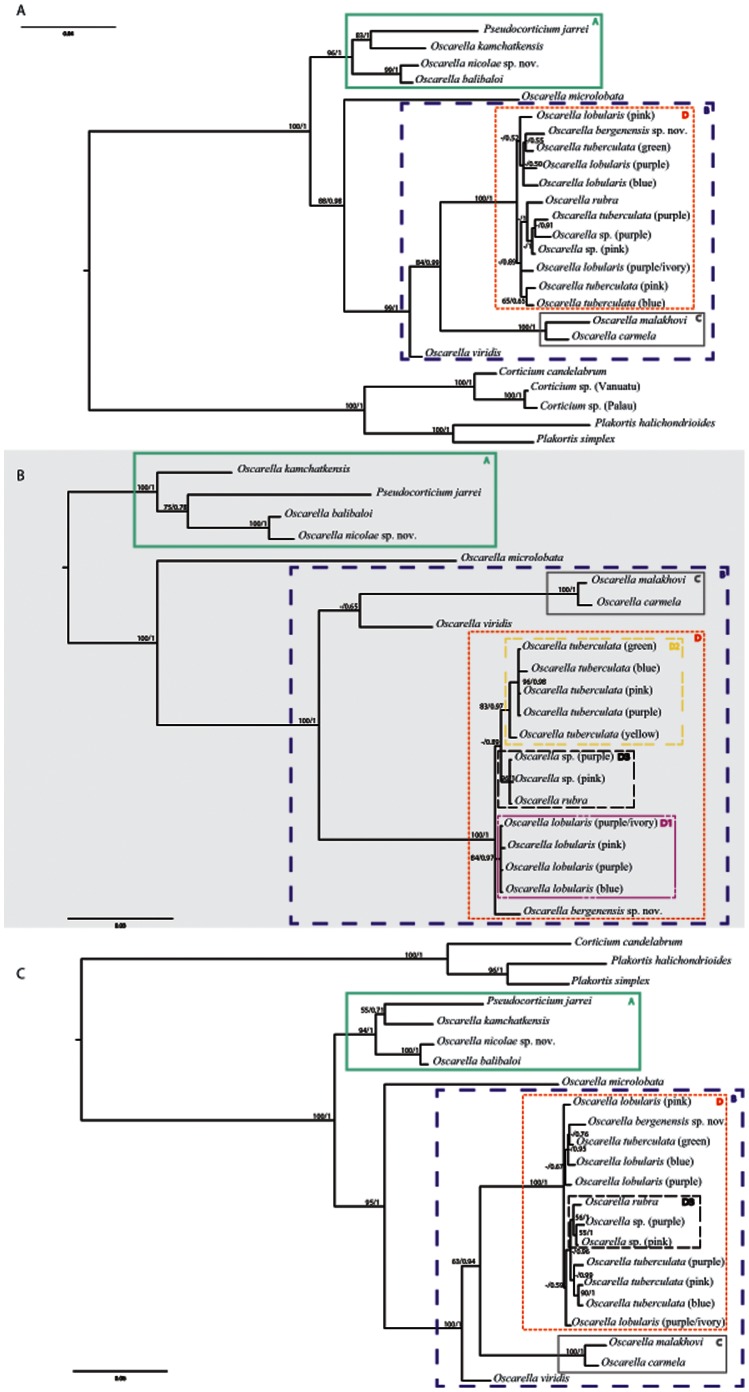
Oscarellidae relationships based on the analyses of concatenated sequences. **(**A) 18S rDNA +28S rDNA nuclear markers, (B) *atp6*+ *tatC* mitochondrial markers and (C) nuclear (18S rDNA +28S rDNA)+mitochondrial (*atp6*+ *tatC*) markers. The topologies presented are posterior consensus trees obtained by the BI analysis using MrBayes. Similar topologies were obtained in ML analysis with PhyML. The numbers are posterior probabilities for BI and bootstrap values (>50) for ML.

The tree topologies obtained using the mt markers are generally congruent with those observed using the nuclear ones (Figures 3 and 4). In the following section, the bootstraps and posterior probability values corresponding to a given node will be listed (in brackets) in this order: (*tatC* BP/PP, *atp6* BP/PP, *tatC*+*atp6* BP/PP). The presence of the four clades (A, B, C, D) defined above is corroborated with the two mitochondrial gene fragments evolving at more rapid substitution rates (Tables 4 and 5). Indeed, all of these clades are strongly supported [A (100/1, 80/0.84, 100/1); B (98/1, 84/0.98, 100/1); C (100/1, 99/1, 100/1); D (100/1, 99/1, 100/1)].

The position of *Oscarella viridis* was resolved as either the sister group of C (in *tatC* tree, Figure 3A) or of D (*atp6* tree, Figure 3B) with moderate support in both cases (65/0.72, 77/0.84, −/0.65). In all cases, the positions observed here are not congruent with those found using nuclear markers.

Furthermore, in contrast to the results obtained with the nuclear genes, both mt markers have partly resolved the relationships between the members of the D clade. The latter can be subdivided into three sub-clades (variably supported): D1, D2 and D3. D1, containing all color morphs of *O. lobularis*, is moderately supported by the analyses of *tatC* and the combined dataset, but is not supported by *atp6* (−/0.71, not found, 84/0.97). D2, grouping all *O. tuberculata* color morphs, is recovered in the analyses of *atp6* (61/0.90) and *tatC*+*atp6* (83/0.97) mitochondrial datasets with moderate support. A sub-clade of *O. tuberculata* - with the exclusion of *O. tuberculata* yellow - received better support in all analyses (93/0.73, 75/0.94, 96/0.98). Finally, D3 groups together *Oscarella* sp. (purple), *O.* sp. (pink) and *O. rubra* (95/1; not found; 96/1). Although the position of the third species from Bergen, *O. bergenensis* sp. nov., was not precisely determined, we noted that with both nuclear and mt markers, it is unrelated to *Oscarella* sp. (pink). The mt combined dataset was more powerful than both nuclear markers and separated mt markers in resolving the relationships among D (Figure 4B).

#### 1.3. Phylogenetic analyses of combined dataset of four markers

The topology obtained from the whole combined dataset is given in Figure 4C. The four main clades (A: 94/1; B: 100/1; C: 100/1; D: 100/1) as well as their interrelationships are retrieved. Among A, as in nuclear topologies, the robust clade [*Oscarella nicolae* sp. nov.+*O. balibaloi*] (100/1) is the sister group of the weakly supported clade [*Pseudocorticium jarrei*+*O. kamchatkensis*] (55/0.71). *O. microlobata* is the sister group of B (95/1) and *O. viridis* is the sister group of D+C (63/0.94) as in 18S rDNA and 18S rDNA +28S rDNA topologies. As in the case for *tatC* marker, the sub-clade D3 is found, its robustness being weakly supported in ML and well supported in BI analyses (56/1).

#### 1.4. Predicted secondary structures of 18S rDNA

**Figure 5:**
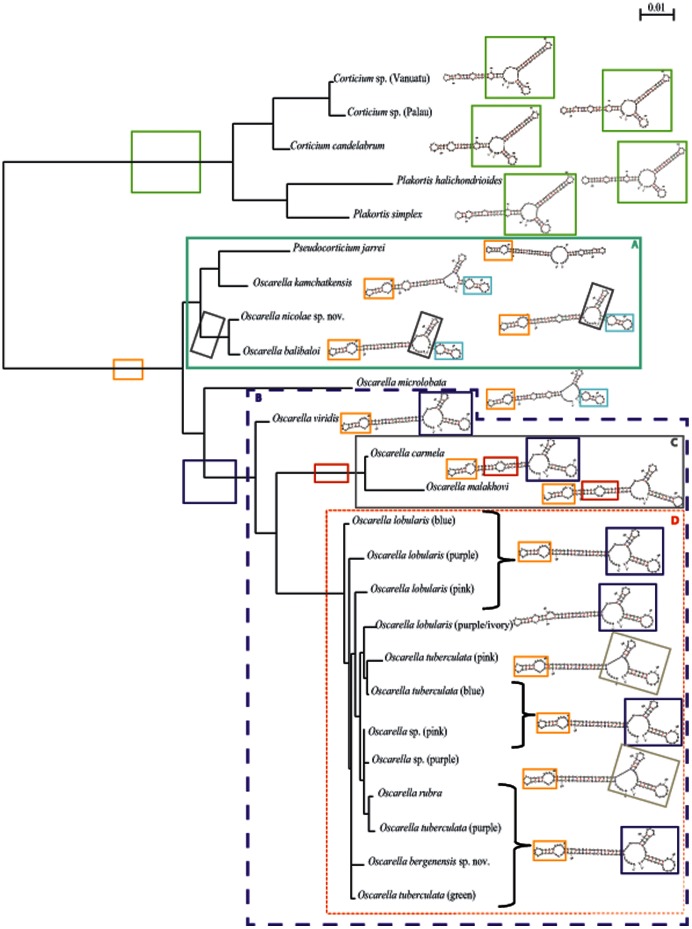
Schemas of the secondary structure predictions of the 18S rDNA V4 region mapped on the 18S rDNA tree topology. Elements composing the structures and included in the characters matrix are circled in a specific color. Characters that can be considered as synapomorphies are mentioned next to the corresponding node.

The predicted secondary structures for the V4 region are presented in Figure 5. We have encoded the molecular morphology of the derived secondary structures as elements (loop, semi-loop and helix) and consider them as binary characters. For this purpose, we use a color code to map them and their appearance. The Oscarellidae species included in this study present different secondary structures, but all except one share a specific element of an internal loop and a terminal loop (in orange, absent in Plakinidae). The presence of this specific structure appears to be diagnostic of Oscarellidae. The absence of this element in *Oscarella lobularis* (purple/ivory) is a derived feature of this morph. Among clade A, *Oscarella nicolae* sp. nov. and *O. balibaloi* share the same secondary structure, with an additional element (compared to the other species of the clade): a central loop plus a helix, which is specific to this group (in black). Most of the species of clade B (C+D+*O. viridis*) share a similar secondary structure with a specific terminal element (one central loop and two helixes, in dark blue). According to the parsimony principle, this element should be considered as a synapomorphy of this group, whereas three species (*O. malakhovi, O.* sp. (purple) and *O. tuberculata* pink) appear to have derived RNA structures. Species from clade C have an extra internal loop lined by three base pairs on each side (in red), which appears to be a synapomorphy of this clade. Three species from A+*O. microlobata* share a common feature (two small terminal loops, in light blue) for which no evolutionary history can be proposed.

#### 1.5. Sequence identity, nucleotide diversity and species delimitation in Oscarellidae

Sequence identity values obtained for the mitochondrial *atp6* and *tatC* sequences for the members of clade D are presented in Table 4. The percentages of identity vary from 99.9 to 100% among *O. lobularis* (D1), from 99.4 to 100% among *O. tuberculata* (D2) and from 98 to 99.5% between the two sibling species. No differences were found for the D3 sub-clade members. *Oscarella bergenensis* sp. nov. is 98.6 to 99.4% similar when compared to the other D members. The *atp6* nucleotide diversity (π) calculation for *O. lobularis* sequences (all color morphs taken together), *O. tuberculata* (all color morphs taken together), *Oscarella* congeneric species (all color morphs for *O. lobularis* and *O. tuberculata* taken together) and D3 sub-clade members are presented in Table 5. Π values for *O. lobularis* (0.000) and *O. tuberculata* (0.001) are low and similar to those obtained for most of Demospongiae while they are five times higher when all sequences from the two congeneric species are taken together.

### 2. Morphological Descriptions and Systematic Justification for Two New Species

According to molecular data, it appears that at least two *Oscarella* samples from Bergen can be considered as distinct species (see discussion), and are thus morphologically and formally described herein.

Phylum PORIFERA Grant, 1836.

Class HOMOSCLEROMORPHA Bergquist, 1978.

Order HOMOSCLEROPHORIDA Dendy, 1905.

Family OSCARELLIDAE Lendenfeld, 1887.

Genus *Oscarella* Vosmaer, 1887.

TYPE SPECIES: *Halisarca lobularis* Schmidt, 1862 (by monotypy). [*Oscaria*] Vosmaer, 1881∶163 (preocc. by *Oscaria* Gray, 1873– Reptilia); *Oscarella* Vosmaer, 1884: pl. 8 (explanation); 1887∶326 (nom. nov. for *Oscaria* Vosmaer). *Octavella* Tuzet and Paris, 1964∶88.

DIAGNOSIS (modified from [57]): Homoscleromorpha without skeleton, with a variable degree of ectosome development. The aquiferous system has a sylleibid-like or leuconoid organization, with eurypylous or diplodal choanocyte chambers. Their mitochondrial genomes encode a gene absent in other animal mitochondrial genomes: *tatC*.

### *Oscarella Bergenensis* sp. nov

TYPE MATERIAL: Holotype: MNHN DJV153, LSID:urn:lsid:zoobank.org:act:2D44BCFA-2163-47C7-9E70-EF6C13E0E4A4, North Sea, Norway, Bergen Fjords, Skarvoysundet +60° 27' 34.74" N, +4° 56' 2.16" E; 3–10 m depth. Collected by Alexander Ereskovsky and Marcin Adamski, 23.06.2009. Paratype: MNHN DJV154. Same data as holotype.

COMPARATIVE MATERIAL EXAMINED: *Oscarella nicolae* sp. nov. (this study), *Oscarella* nathaliae Ereskovsky, Lavrov, Willenz, 2013 (RBINS POR 90, RBINS POR 91, RBINS POR 92 and RBINS POR 94: Caribbean Sea: S Martinique, N Jamaica, Guadeloupe) [18]. *Oscarella malakhovi* Ereskovsky, 2006 (ZIN RAS 10697 ZIN RAS 10698: Japan Sea) [15]. *Oscarella kamchatkensis* Ereskovsky, Sanamyan & Vishnyakov, 2009 (ZIN RAS 11058, ZIN RAS 11059 and ZIN RAS 11060: East-North Pacific, Avacha Gulf) [16]. *Oscarella lobularis* (Schmidt, 1862) [10], *Oscarella tuberculata* (Schmidt, 1868), NW Mediterranean Sea (Marseille region). *Oscarella microlobata* Muricy, Boury-Esnault, Bézac, Vacelet, 1996 [30], *Oscarella viridis* Muricy, Boury-Esnault, Bézac, Vacelet, 1996 [30], *Oscarella balibaloi,* Pérez, Ivanišević, Dubois, Pedel, Thomas, Tokina, Ereskovsky, 2011 [17] NW Mediterranean Sea (Marseille region).

DIAGNOSIS: Red-orange *Oscarella* at the apical parts and patchy yellow at the basal parts, with folded surface and average resilience consistency; containing two particular cell types with inclusions (vacuolar and granular cells), archaeocytes in low number in the mesohyl and one morphotype of endobiontic bacteria.

**Figure 6:**
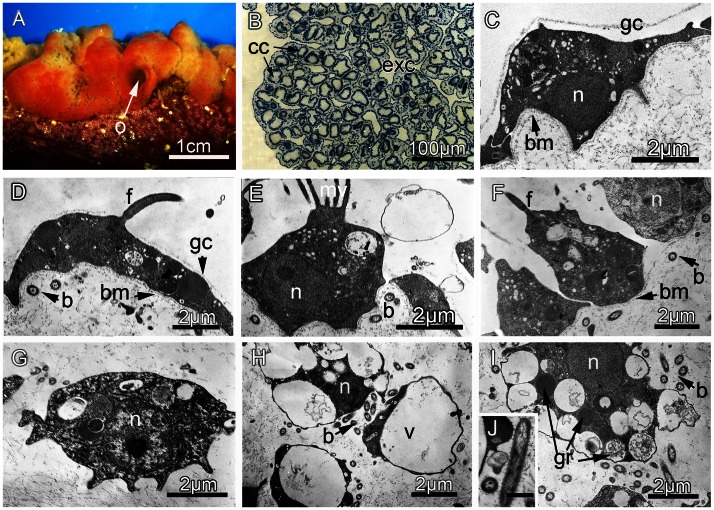
*Oscarella bergenensis* sp. nov. (A) External morphology *in vivo*. (B) General anatomy, observed with light microscopy. (C) TEM of exopinacocyte. (D) TEM of endopinacocyte. (E) TEM of apopylar cell. (F) TEM of choanocyte. (G) TEM of archaeocyte. (H) TEM of vacuolar cells. (I) TEM of granular cells. (J) TEM of symbiotic bacteria. (b) Symbiotic bacteria; (bm) Basement membrane; (cc) Choanocyte chamber; (ec) Ectosome; (exc) Exhalant canal; (exp) Exopinacodem; (f) Flagellum; (gc) Glycocalyx; (gr) Granules; (mv) Microvilli; (n) Nucleus; (o) Osculum; (v) Vacuole. Scale bar: J = 0.5 µm.

DESCRIPTION: Moderately large, encrusting, size from 2x1 cm to 6x4 cm, thickness 4–8 mm. Easy to detach from its substrate. Smooth surface but with small folds. Oscula at the end of small conical lobes 2–3 mm in height, not transparent. Consistency: average resilience. Color *in vivo* red-orange at the apical parts of sponge and patches of yellow and orange in inner parts, not bright (Figure 6A).

SOFT TISSUE ORGANISATION: Spicule and fiber skeleton absent. Ectosome from 9 to 20 µm thick (Figure 6B). Inhalant canals (12 µm in diameter) running perpendicular to the surface (Figure 6B). Choanocyte chambers are eurypylous, roughly spherical to ovoid, about 32 µm in diameter (Figure 6B). Choanocyte chambers are arranged around inhalant and exhalant canals in a sylleibid pattern. Exhalant canals about 40 µm in diameter running toward well-developed system of basal cavities, leading to the oscula. Ostia regularly distributed, 16–21 µm in diameter.

CYTOLOGY: Exopinacocytes (Figure 6C) are flat (7.1 µm wide by 1.9 µm high), flagellated. Nucleus is ovoid (1.6 µm in diameter), often with a visible nucleolus. Cytoplasm contains inclusions, phagosomes (from 0.2 to 0.8 µm in diameter) and vacuoles (0.1–0.9 µm in diameter). Endopinacocytes (Figure 6D) are flat (7.9 µm wide by 2.4 µm high), flagellated, often with thin cytoplasm projections in their basal part. Nucleus is ovoid (2.2 µm). Cytoplasm contains numerous osmiophilic inclusions as well as phagosomes (from 0.3 to 0.9 µm in diameter) and vacuoles (0.1–1.1 µm in diameter). Apopylar cells (Figure 6E) are triangular in section (5.2 µm wide and 3.2 µm high). They are flagellated with a crest of microvilli. Nucleolated nucleus is basal, ovoid, up to 2 µm in diameter. Cytoplasm contains phagosomes and small osmiophilic inclusions. Choanocytes have irregular, pyramidal to trapeziform shape (3.4–4.7 µm wide and 4.9–6.1 µm high) (Figure 6F). Nucleus is central or basal, ovoid (about 2.1 µm in dimension), often with a nucleolus. Cytoplasm usually contains phagosomes (0.5–1.5 µm in diameter) and electron transparent vacuoles (0.18–1.0 µm). The adjacent choanocytes are in contact with each other at their basal parts. A thin, irregular layer of glycocalyx covers the surface of exopinacocytes, endopinacocytes, choanocytes and apopylar cells. Choanoderm and pinacoderm are lined with a basement membrane, which is a continuous, 15–25 nm thick layer of condensed collagen microfibrils (Figure 6C, D, F).Archaeocytes (Figure 6G) are amoeboid, (5.6 µm wide by 3.3 µm length), dispersed in low number in the mesohyl. Cytoplasm includes small phagosomes, rare electron transparent vacuoles from 0.2 to 1.1 µm in diameter and rare osmiophilic inclusions, from 0.2 to 0.8 µm in diameter. A large nucleolated nucleus is spherical or ovoid, 2.2 µm in diameter.Two types of cells with inclusions are numerous within the mesohyl: Vacuolar cells (Figure 6H) are ovoid to irregular, 6.4 to 8.8 µm long with ovoid or slightly irregular nucleus 1.9 µm in diameter without nucleolus. Their cytoplasm has one to four large, irregular vacuoles (about 1.1×1.6 µm to 4.1×5.2 µm) with clear, filamentous contents. Vacuoles are often brought together. Granular cells (Figure 6I) are ovoid to irregular (7.8 µm long and about 4.4 µm width). The nucleus is 2.2 µm in diameter. Cytoplasm is filled with 10 to 16 electron-transparent spherical vacuoles about 0.8 µm in diameter with loosely dispersed material. Rare electron-dense homogenous granules (0.4–0.9 µm in diameter) are present in the cytoplasm. Symbiotic bacteria belonging to a single morphotype are dispersed extracellularly in the mesohyl (Figure 6J). They are elongated, rod-like (length 1.1–1.8 µm and diameter 0.25–0.35 µm). The cell wall is Gram-negative and consists of two layers. A filamentous network of the nucleoid is irregular with thick elements in the center and thin filaments closer to the periphery of the cell. A small layer of granular cytoplasm is observed near the cytoplasm membrane. Short, radially-arranged filaments are present outside the cytoplasmic membrane.

REPRODUCTION: Only rare early oocytes (before vitellogenesis) were observed in the material (collected from mid- to late June).

HABITAT: Depth 3–10 m; abundant as epiphyte on the basal parts of thalli of *Laminaria digitata*, on granite rock, vertical walls.

ETYMOLOGY: The species name is derived from the site where it was discovered (Bergen fjords).

TAXONOMIC REMARKS: Regarding color, *Oscarella bergenensis* sp. nov. is unusual: red-orange at the apical parts with patches of yellow at the inner parts. This coloring is almost unique compared to all described *Oscarella* species: only some specimens of *O. rubra* [58] from Roscoff have the same color pattern (AE, personal observation). The surface of *O. bergenensis* sp. nov. is smooth with small folds, and is thus similar to that of *O. rubra.* Cells with inclusions of the mesohyl are important discriminating characters in *Oscarella* taxonomy. These cells are diverse and abundant in *Oscarella* species, each species being characterized by a distinctive set of cells with highly variable inclusion types [12], [15], [16], [17], [29], [59]. The total cell composition and ultrastructure of *O. bergenensis* sp. nov. differs from all other Mediterranean and Pacific *Oscarella* species (Table 3). *O. bergenensis* sp. nov. does have a vacuolar cell very similar to those of *O. microlobata, O. carmela* and *O. balibaloi* [17], [29], [30]. However, the granular cells of *O. bergenensis* sp. nov. are quite different from the cells of this type described in *O. microlobata,* and *O. balibaloi,* and also in *O. carmela, O. malakhovi, O. viridis* and *O. ( = Pseudocorticium) jarrei. O. bergenensis* sp. nov. shares the presence of archaeocytes with *O. viridis, O. carmela, O. malakhovi, O. tuberculata* and *O. nicolae* sp. nov.

### *Oscarella Nicolae* sp. nov

TYPE MATERIAL: Holotype: MNHN DJV155, LSID urn:lsid:zoobank.org:act:DFFAD94B-9CAD-4F99-994D-BDEF75EF98A1, North Sea, Norway, Bergen Fjords, Skarvoysundet +60° 27' 34.74" N, +4° 56' 2.16" E; 3–10 m depth. Collected by Alexander Ereskovsky and Marcin Adamski, 23.06.2009. Paratype: MNHN DJV156. Same data as holotype.

COMPARATIVE MATERIAL EXAMINED: *Oscarella bergenensis* sp. nov. (this study), *Oscarella* nathaliae Ereskovsky, Lavrov, Willenz, 2013 (RBINS POR 90, RBINS POR 91, RBINS POR 92 and RBINS POR 94: Caribbean Sea: S Martinique, N Jamaica, Guadeloupe) [18]. *Oscarella malakhovi* Ereskovsky, 2006 (ZIN RAS 10697 ZIN RAS 10698: Japan Sea) [15]. *Oscarella kamchatkensis* Ereskovsky, Sanamyan & Vishnyakov, 2009 (ZIN RAS 11058, ZIN RAS 11059 and ZIN RAS 11060: North Pacific, Avacha Gulf) [16]. *Oscarella lobularis* (Schmidt, 1862) [10], *Oscarella tuberculata* (Schmidt, 1868), NW Mediterranean Sea (Marseille region). *Oscarella microlobata* Muricy, Boury-Esnault, Bézac, Vacelet, 1996 [30], *Oscarella viridis* Muricy, Boury-Esnault, Bézac, Vacelet, 1996 [30], *Oscarella balibaloi,* Pérez, Ivanišević, Dubois, Pedel, Thomas, Tokina, Ereskovsky, 2011 [17] NW Mediterranean Sea (Marseille region).

DIAGNOSIS: Thinly encrusting *Oscarella*, ivory-yellowish, not bright in colour, with microlobate surface and soft, delicate, slimy consistency and abundance of mucus; containing two distinct cell types with inclusions (granular cells and spherulous cells with paracrystalline inclusions), abundant archaeocytes and one morphotype of endobiontic bacteria.

**Figure 7:**
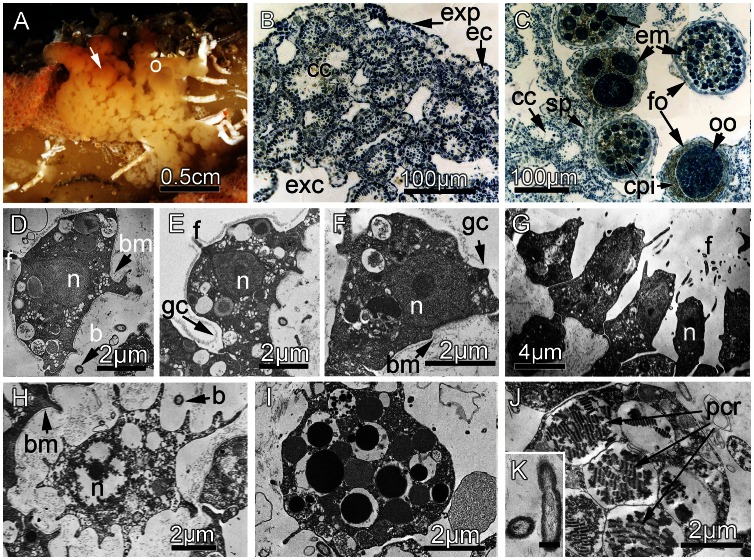
*Oscarella nicolae* sp. nov. (A) External morphology *in vivo*. (B) General anatomy, observed with light microscopy. (C) Light microscopy, details of hermaphrodite sponge during reproduction. (D) TEM of exopinacocyte. (E) TEM of endopinacocyte. (F) TEM of apopylar cell. (G) TEM of choanocyte. (H) TEM of archaeocyte. (I) TEM of granular cell. (J) TEM of spherulous cells with paracrystalline inclusions. (K) TEM of symbiotic bacteria. (b) Symbiotic bacteria; (bm) Basement membrane; (cc) Choanocyte chamber; (cpi) Spherulous cells with paracrystalline inclusions; (ec) Ectosome; (em) Embryos; (exc) Exhalant canal; (exp) Exopinacoderm; (f) Flagellum; (fo) Follicle; (gc) Glycocalyx; (gr) Granules; (n) Nucleus; (o) Osculum; (oo) Oocyte; (pcr) Spherules with paracrystalline inclusions; (sp) Spermatic cyst. Scale bar: J = 0.5 µm.

DESCRIPTION: A thinly encrusting sponge, covering surface areas up to 2 cm^2^ in area to thicknesses of 1.5–3 mm (Figure 7A). The sponge is very difficult to detach from the substratum. The surface is microlobate. Oscula are at the end of cylindrical tubes up to 3 mm high, transparent. Consistency: not resilient, very soft and fragile. Abundance of mucus. Color *in vivo* ivory-yellowish, not bright.

SOFT TISSUE ORGANISATION: Sponge lacks spicule and fiber skeleton. Ectosome from 10 to 20 µm thick. Inhalant canals with diameter about 25 µm run perpendicular to the surface (Figure 7B). Exhalant canals run toward a system of basal cavities, leading to the oscula. Choanocyte chambers ovoid to spherical, eurypilous about 50 µm in diameter (Figure 7B, C). The aquiferous system has a sylleibid-like organization. Ostia are 8–12 µm in diameter and regularly distributed.

CYTOLOGY: Exopinacocytes (Figure 7D) are flat to lens-like, flagellated, about 7.4 µm wide by 3.2 µm high. They are anchored in the mesohyl by long thin basal pseudopodia. Nucleus is ovoid (2.3 µm in diameter), often with a nucleolus. Cytoplasm contains inclusions and phagosomes from 0.4 to 1.3 µm in diameter. Endopinacocytes (Figure 7E) are flat, flagellated, about 9.2 µm wide by 2.3 µm high, often showing irregular, thin cytoplasm projections in their basal part. Nucleus is ovoid (1.6×2.7 µm). Cytoplasm contains numerous osmiophilic inclusions and phagosomes 0.6–1.1 µm in diameter.

Apopylar cells (Figure 7F) are roughly triangular in section (7.5–9.2 µm high and 4.2–5.7 µm wide). The cells have small lateral pseudopodia. The nucleus is nucleolated, apical, ovoid, up to 2.2×3 µm in cross-section. Cytoplasm contains phagosomes and small osmiophilic inclusions. Choanocytes have irregular, pyramidal to prismatic cell bodies (4.6 µm wide at the central part and 9.1 µm high) (Figure 7G). Nucleus is central or apical, ovoid (2.1×3.9 µm in dimension), often with a nucleolus. Cytoplasm usually contains phagosomes (0.6–1.6 µm in diameter), smaller digestive vacuoles and osmiophilic inclusions. The adjacent choanocytes are in contact with each other at their central or basal parts. The cells have short pseudopodia. Choanoderm and pinacoderm are lined with a basement membrane, which is a continuous, 12–19 nm thick layer of condensed collagen microfibrils (Figure 7D, E, F). A thin, irregular layer of glycocalyx covers the surface of exopinacocytes, endopinacocytes, choanocytes and apopylar cells. Archaeocytes (Figure 7H) are amoeboid (7.7 µm wide by 5.1 µm length). A large nucleolated nucleus is spherical or ovoid (3.8×2.9 µm). Cytoplasm includes small phagosomes and rare electron-transparent vacuoles (from 0.6 to 1.6 µm in diameter). Two types of cells with inclusions occur within the mesohyl: (i) Granular cells (Figure 7I): ovoid, 7.4 µm long and about 5.9 µm in diameter. The nucleus is 2.3 µm in diameter. Cytoplasm is filled with 6 to 12 electron dense homogenous granules, 0.6–1.2 µm in diameter. In the cytoplasm, there are some electron-transparent vacuoles (0.5–2.2 µm in diameter). Other special inclusions are absent. (ii) Spherulous cells with paracrystalline inclusions (Figure 7J): ovoid or rarely spherical cells 7.1 µm long and 5.5 µm in diameter with nucleolated nucleus 2.2 µm in diameter. Cytoplasm is filled with 5–9 spherical heterogeneous inclusions (0.9–3.2 µm in diameter), composed of paracrystalline elements included in a homogenous matrix. Paracrystalline elements are ovoid or cylindrical in longitudinal section and round in transversal section (0.7 µm long and 0.35 µm in diameter). These elements are composed of fibrils arranged in a transverse banding pattern with dark bands alternated by clear bands. In cross sections, the paracrystalline elements are organized in spiral lines. Cytoplasm can also contain 4–8 spherical granules (0.5–1.1 µm) with electron-dense homogenous inclusions and electron transparent vacuoles (0.7–1.7 µm in diameter). These cells concentrate around the eggs and are located inside them after the closing of follicle (Figure 7C). The cells are present during embryogenesis inside the embryos (Figure 7C). Symbiotic bacteria: One morphological type of endosymbiont, extracellular bacteria occurs in the mesohyl (Figure 7D, H, K). This type of bacteria is elongated to oval (0.8–1.5 µm long and 0.37–0.8 µm in diameter). Under the cell wall a layer of dense filaments can be observed. The nucleoid zone consists of an almost regular filamentous network. Some bacteria have internal mesosome-like structures. A developed glycocalyx is present at the surface of bacteria.

REPRODUCTION: *Oscarella nicolae* sp. nov. is ovoviviparous and simultaneously hermaphrodite: in the same individuals male and female reproductive elements are present. The spermatic cysts have different diameters (from 20 to 95 µm) and are randomly distributed in the sponge mesohyl (Figure 7C). Spermatogenesis is generally asynchronous inside spermatic cysts. Oogenesis and embryogenesis are asynchronous, all stages from oogonia to egg were observed within the same specimen. Mature eggs are isolecithal and polylecithal, with a cytoplasm full of yolk granules (Figure 7C). Embryogenesis is also asynchronous. All stages from cleaving embryos to prelarva were observed from mid-June to mid-July (Figure 7C). Eggs and embryos are located in the basal part of the choanosome and are completely surrounded by a follicle made of endopinacocytes.

HABITAT: Depth 3–10 m, common and abundant as epiphyte on the basal parts of thalli of *Laminaria digitata*, on granite rock, vertical walls.

ETYMOLOGY: This species is named in honour of Dr Nicole Boury-Esnault, a remarkable taxonomist and biologist of sponges, who first drew the attention to the great diversity of *Oscarella*.

TAXONOMIC REMARKS: The ivory-yellowish color of *Oscarella nicolae* sp. nov. is not unique, but very rare in other *Oscarella*. For example, some individuals of *O. balibaloi* and *O. tuberculata* from the Mediterranean Sea can also display this color. *O. nicolae* sp. nov. shares its soft consistency with *O. viridis* and *O. balibaloi*, and the aspect of its surface with *O. microlobata, O. kamchatkensis* and *O. balibaloi*. The soft slimy delicate consistency with abundant mucus, characteristic of *O*. *nicolae* sp. nov. differs significantly from *O. bergenensis* sp. nov. The cell composition and ultrastructure of *O. nicolae* sp. nov. differ from all other Mediterranean and Pacific *Oscarella* species (Table 3). Among the two secretory cell types of the mesohyl of *O. nicolae* sp. nov., the spherulous cells with paracrystalline inclusions are very similar to the spherulous cells of *O. ( = Pseudocorticium) jarrei, O. microlobata* (type II), *O. imperialis* (type I), *O. kamchatkensis* (type I) and *O. balibaloi* (type 1) [7], [16], [17], [30]. The granular cells of *O. nicolae* sp. nov. are similar to those of *O. kamchatkensis.* This species shares the presence of archaeocytes with *O. viridis, O. carmela, O. malakhovi, O. tuberculata* and *O. bergenensis* sp. nov. One of the important differences between the two new species is their period of reproduction: *O. bergenensis* sp. nov. contained only young oocytes in mid-June to mid-July, whereas *O. nicolae* sp. nov. is hermaphroditic with simultaneous gametogenesis (oogenesis and spermatogenesis) and embryogenesis during the same period.

### 3. Evolutionary Histories of Non-molecular Characters

The ecological, morphological, cytological, and embryological characters of Oscarellidae species examined in this study are presented in Tables 2 and 3.

Some of the cytological characters mentioned in these tables have often been mislabeled in previous publications and need to be clarified. According to the Thesaurus of Sponge Morphology [60] spherulous cells are cells filled with large round spherules that occupy almost the entire cytoplasm. Unfortunately, *Oscarella'*s cytology descriptions in publications are sometime ambiguous. Indeed, the cell type designations used are confusing especially concerning granular, spherulous and globular cell terms. For our analysis, we did a complete revision of previously published descriptions and photos of *Oscarella* spp., as well as a comparative analysis of TEM pictures from our collection. The results of this analysis are provided in Table 3. Spherulous cell type was described in six *Oscarella* spp.: *O. balibaloi, O. imperialis, O. kamchatkensis, O. microlobata, O. ( = Pseudocorticium) jarrei and O. nicolae* sp. nov. [7], [16], [17], [30]. In all these species, spherulous cells have characteristic paracrystalline inclusions which have never been described in other homoscleromorphs and which are very uncommon in other sponges. Furthermore, one additional type of spherulous cell "with granular inclusions" and without paracrystalline elements was described in *O. microlobata* [30].

Evolutionary histories of characters were examined and some were found to be not informative, as they were constant in all sampled specimens (Figure S2A). This is the case for the basement membrane and several embryological characters that are present in all Homoscleromorpha. Other characters are highly variable in each species, and thus contain little phylogenetic signal. None of the recorded ecological, geographical and external morphological characters reflects phylogenetic relationships of *Oscarella* species. Furthermore, no color, type of consistency or sort of surface can be linked to any molecular clade. Nine histological and cytological characters were compared. It is noteworthy that one cytological character, the presence of spherulous cells with paracrystalline inclusions, is found in clade A species and also in *O. microlobata* which has an unclear position in phylogenetic analyses (Figure S2B). Three characters, concerning the aquiferous system and the cortex, are constant for all but one species: *Pseudocorticium jarrei,* now *Oscarella jarrei,* which has character-states similar to the Plakinidae (outgroup) (Figure S2C). The sylleibid aquiferous system and the eurypylous choanocyte chambers appear diagnostic for Oscarellidae, however, these characters have also been described in some *Plakina* species (Plakinidae): *P. trilopha, P. monolopha*, *P. crypta*, *P. endoumensis, P. jani* [61]. In addition, we also looked at four other cellular types (archaeocytes, vacuolar cells, granular cells and spherulous cells). For the most part, their absence or presence in different states cannot be related to any relationship supported by molecular data (Figure S2D). Nevertheless, we noticed that the vacuolar cells are absent in all clade A species with the exception of *O. balibaloi*. Our analysis suggests that the ancestral state for A members is the “absence” of vacuolar cells and that the state of *O. balibaloi* is due to reversal. In conclusion, we found very few morphological and cytological diagnostic characters supporting the clades defined based on molecular data. Clade A appears to be characterized by the absence of vacuolar cells and the presence of spherulous cells with paracrystalline inclusions, but with the exceptions of *O. balibaloi* (the member of the clade having vacuolar cells) and of *O. microlobata* (outside clade A - although with a poorly defined position - and having spherulous cells with paracrystals).

## Discussion

### 1. Suitability and Limits of Molecular Markers Used

All four markers used for this study were informative for understanding phylogenetic relationships within Oscarellidae and produced mostly congruent trees (Figures 2, 3 and 4). However, some differences were observed in the performance of individual markers and are discussed below.

The 18S rDNA analyses resolved the deeper nodes of the phylogenies. The suitability of this marker for reconstructing relationships at family level has often been demonstrated in sponge phylogenies [27]. Nevertheless, as previously noticed, its power of resolution is insufficient for deciphering relationships between closely-related species: this is the case here - and was expected given our previous data - for *Oscarella tuberculata* and *O. lobularis* color morphs, and more generally within the clade D. The 28S rDNA mostly confirms the main topology obtained with the 18S rDNA but did not provide higher resolution for the D clade. Moreover, the inferred relationships within D were different between the two nuclear markers. These observed discrepancies could be explained either by scarcity of the phylogenetic signal or by the fact that these markers constitute multigene families. Although it is generally assumed that paralogous copies of rDNA genes are homogeneous [62], [63], [64] because they evolve by concerted evolution, this is not always the case, and conflicting phylogenies have been inferred when using different copies of ITS [65], [66] or 18S rDNA [67], [68]. Nevertheless, one can suppose that the action of concerted evolution may not be sufficient to compensate paralogous evolution for recently diverged species.

Mitochondrial markers provide several advantages for phylogenetic reconstructions including their higher rate of sequence evolution and the rarity of gene duplication. Assuming the uniparental inheritance (common for most metazoans) and the effective absence of recombination (characteristic for Metazoa), the whole mtDNA should evolve as a single locus.

Thus, we selected two regions that showed the highest diversity in Oscarellidae in our preliminary results. Not surprisingly, these two regions did not include *cox1*, which is one of the most conserved regions of the mitochondrial genome and often fails to resolve relationships among closely-related sponge species [69], [70] and, in general, often performs worse in resolving animal relationships than other genes. The *atp6* marker has already been selected for alpha-level systematics in Demospongiae because it was more polymorphic than *cox1* in the studied species [38]. In our case, despite comparable nucleotide diversity (Π, Table 5), *atp6* appears not to be powerful enough to clearly resolve phylogenetic relationships among members of D clade. *TatC* is the gene that encodes the subunit C of twin-arginine translocase. The Twin-Arginine Translocation Pathway is involved in transfer of folded proteins across biological membranes in bacteria, chloroplast and, possibly, mitochondria [71]. *TatC* gene is not found in any other sponge or animal mtDNA [72], [73] but is commonly present in mitochondrial genomes of other eukaryotes [74]. It was first reported in the *O. carmela* genome [73] and then found in other Oscarellidae species ([6], this study). This gene appears to be more variable than *atp6* and is the most variable of our four markers. The *tatC* marker thus helped to better resolve the D clade relationships and led us to propose the D1, D2 and D3 sub-clades. We were surprised that despite their geographical distance and cyto-morphological differences, the three samples of sub-clade D3 have identical sequences for both *tatC* and *atp6* genes. This observation suggests either the presence of three different morphotypes of only one species (see section 4.4) or the fact that both mt markers are non-resolving at this scale. The limit of resolution issue for mt markers was already known for non bilaterian species [75]. A study at a finer scale using more variable markers (e.g. introns) will be necessary in the future to understand the evolutionary history of D3 individuals.

### 2. Monophyly of *Oscarella* and Synonymy with *Pseudocorticium*

Our results confirm that the Oscarellidae, i.e. the Homoscleromorpha without skeletons, are separated into two clades A and B. *Pseudocorticium jarrei* is clearly included in clade A, and this is congruent with chemical data obtained by metabolic fingerprints [8]. The species also shares with all but one *Oscarella* spp. a character of the secondary structure of the 18S rDNA V4 region (internal and terminal loop), which can be considered as diagnostic for this group. *Pseudocorticium jarrei*, however, differs from *Oscarella* spp. by the development of the mesohyl, the aquiferous system and the thickness of the cortex. Two taxonomic interpretations are possible: (i) to maintain the genus *Pseudocorticium* for clade A, with transfer of *Oscarella balibaloi*, *O. nicolae* sp. nov. and *O. kamchatkensis* to *Pseudocorticium*, (ii) to synonymize *Pseudocorticium* with *Oscarella*, with transfer of *P. jarrei* to *Oscarella*. We prefer the second alternative involving fewer changes, considering that the outer morphology, aquiferous system and development of the mesohyl of *P. jarrei* can be considered as homoplasic features [6]. We thus propose the abandonment of the genus *Pseudocorticium*, which is presently made up of only one species. *Pseudocorticium jarrei* is thus transferred to *Oscarella*. Consequently, the Oscarellidae becomes a monogeneric (*Oscarella*) family, with the following definition: Homoscleromorpha without skeletons, with a variable degree of ectosome development. The aquiferous system has a sylleibid-like or leuconoid organization, with eurypylous or diplodal choanocyte chambers. Their mitochondrial genomes encode a gene absent in other animal mitochondrial genomes: *tatC* [6].

### 3. The Two Sibling Species *Oscarella Lobularis* and *O. tuberculata*

Our molecular results, including several color morphs for both *Oscarella lobularis* and *O. tuberculata,* mainly confirm and extend the previous study based on allozymes [12]. Indeed, phylogenetic analyses, molecular divergence and nucleotide diversity calculations suggest that *O. lobularis* and *O. tuberculata* are two different and distinct species that live in sympatry. This is also corroborated by the presence of diagnostic positions in both mt DNA markers for each species (Figure S3). Cell composition [12], symbiotic microbe diversity [21], as well as life history traits such as sex ratios and reproductive cycles, also differ between *O. lobularis* and *O. tuberculata,* consistent with the presence of two distinct species [14]. Concerning color polymorphism, the type species *O. lobularis* displays not only the classical purple/ivory color arrangement but can also be blue, pink and purple. *O. tuberculata* specimens also present a variety of colors from yellow to purple. The high polychromism observed in these two species is quite unusual, compared to other sponges, although color variability is often observed between specimens of sponges living in different local environmental conditions (shady side *vs* exposed to light) [76] and non-ecophenotypical polychromism in sponges can also be found (e.g. *Mycale* species [77]). All color morphs of both *O. lobularis* and *O. tuberculata* from our study live in the same areas and environments. A wide comparison within sponges is hampered by the fact that very few studies combining molecular and morphological data on color morphs of sponges have been conducted so far.

In several genera, it has been shown that color morphs are, in fact, distinct species: e.g. *Suberites ficus* from Great Britain using allozymes [78], or *Latrunculia* from New Zealand, using allozymes and chemical studies [79]. An almost comparable situation to what we found in *Oscarella* species is the case of *Callyspongia vaginalis*. This species has three morphotypes that vary in both color and shape, but which have identical sequences for four molecular markers, thus revealing that they all belong to the same species [80] (or that the markers do not evolve fast enough).

We cannot unequivocally exclude that each color morph (for both *O. tuberculata* and *O. lobularis*) may represent populations in the course of speciation; each might become a new species in the future. This is particularly the case of the *O. tuberculata* yellow morph, which is the most divergent in this species. In addition, these color morphs may also already be different recent species that were not discriminated by the markers used. A population genetics study using more variable markers with more specimens of various localities is needed to conclusively solve this question (assuming that “true” species exist in nature: see [81] for some insights). Nevertheless, the absence of distinguishable morphological characters between these color morphs, and particularly the fact that they are sympatric has led us now to prioritize, the polychromism hypothesis, and hence the presence of only two species.

As the homoscleromorph sponge *O. lobularis* is now emerging as a sponge model for evo-devo studies [13], [22], [23], [24], clear criteria for sampling are indispensable. As color is not a valid diagnostic character to distinguish this sponge from *O. tuberculata*, only the *in situ* evaluation of the specimen consistency (soft *vs* cartilaginous) is useful during collection. This could be complemented by sequencing *atp6* and/or *tatC* and looking for diagnostic positions (see Figure S3) and for the presence of vacuolar cells in LM.

### 4. One or Several Species in sub-clade D3? An Open Question

Our results based on phylogenetic analyses, molecular divergence and nucleotide diversity calculations have revealed no divergence/diversity between *Oscarella* sp. (purple), *O. rubra* and *O.* sp. (pink) in mitochondrial sequences. However, conventional taxonomy based on morphological characters has revealed that these samples have very distinct features (such as outer morphology, cytology and microbial composition, see Tables 2 and 3). These three specimens could represent a single, morphologically variable species, *Oscarella rubra,* or may represent three distinct species. This can be resolved only by a more complete study based on more numerous specimens.

### 5. At least Two New *Oscarella* Species from Bergen Fjords

In this study, three different species/specimens of sponge, living in sympatry and belonging to the *Oscarella* have been sampled in Bergen fjords. Molecular data, especially the mitochondrial data, have revealed that these specimens are not closely-related but belong to three distinct species. Consequently, two of them have been morphologically described and formally named (see results). *Oscarella nicolae* sp. nov. belongs to clade A and is more closely-related to *O. jarrei*, *O. kamchatkensis* and *O. balibaloi* than to the other species from Bergen fjords. Its external morphology and, in particular, its microlobate surface, distinguish this species from the two others. Cytological characters are also informative: archaeocytes are often present in its mesohyl, while no vacuolar cells have been found, contrary to the two other species. Moreover, *O. nicolae* sp. nov. contains spherulous cells with paracrystalline inclusions and has no vacuolar cells as is the case for the other members of clade A. The affinity of *O. nicolae* sp. nov. with *O. balibaloi* is also supported by a shared secondary structure element in their 18S rDNA. *Oscarella bergenensis* sp. nov. is included in clade D, with uncertain relationships regarding D1, D2 and D3 sub-clades. It is clearly distinct from the two other samples from Bergen, as shown by phylogenetic analyses. Furthermore, its external morphology (color and consistency) is different to the two other specimens from Bergen, while its histological and cytological features are quite similar to *Oscarella* sp. (pink). The last Bergen specimen, *Oscarella.* sp. (pink) belongs to D3 sub-clade and it is unclear whether it is *O. rubra* or a new species (see 4.4). Present data are insufficient, preventing firm conclusions from being drawn, as a result, we propose no morphological description for the moment.

### Conclusions

**Figure 8:**
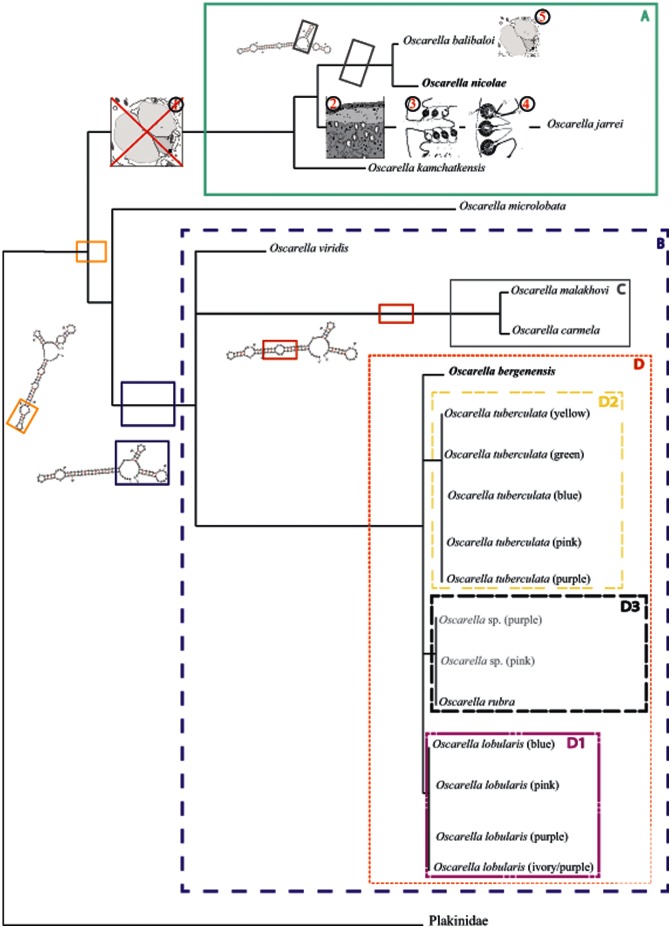
Simplified consensus tree based on *tatC*+*atp6* molecular phylogenies. All robust nodes (BP>50+ PP>0.5) were conserved. Polytomy was prioritized for weakly-supported nodes (BP<50 or PP<0.5). Molecular and non-molecular characters that are synapomorphies/diagnostic characters of the clades are indicated on the corresponding nodes. The absence of vacuolar cells (1) is diagnostic of A. The presence of cortex (2), leuconoid aquiferous system (3) and diplodal chambers (4) are specific characters of *Oscarella jarrei*. The presence of vacuolar cells in *O. balibaloi* is a reversal character (5) The two new species names are indicated in bold in the tree. In light grey are specimen/species for which uncertainties remain (new species or not). Schemas of morphological characters are modified from [60] or are new.

The taxonomy of sponges is usually based on the characters of the skeleton, fibers and spicules. Due to the absence of skeleton and to the presence of mainly invariable histological character-states, the identification of *Oscarella* at the species level is very difficult. The differences among species are mostly in external traits: color, consistency, and aspect of the surface [12], [15], [16], [17], [18], [28], [29], [57], but these characters must be evaluated with care; they can be highly subjective and also very polymorphic. At the same time, some cytological characters, such as the presence of types of cells with inclusions and symbiotic microbes morphology, have been proposed to be more informative for some *Oscarella* species identification [15], [16], [17], [18], [21], [29]. These findings are corroborated by the present molecular study because species previously described using morphology are confirmed here with molecular data. Nevertheless, except for two (absence of vacuolar cells and possibly spherulous cells with paracrystalline inclusions), these morphological character-states are not powerful tools for reconstructing species relationships in this group (Figure 8). Mapping morphological characters on molecular trees to identify synapomorphies which support clades has been a successful approach for diverse groups (e.g. Demospongiae, Calcarea [2], [34], [82], [83]) although it has also been poorly indicative or unsuccessful for some demosponge taxa: Haplosclerida, Halichondrida, Axinellidae [37], [84], [85], [86], as well as for Oscarellidae (Figure 8). This study offers additional evidence that when there are few available morphological features to study and compare, molecular biology offers a powerful tool to provide insights into phylogenetic relationships [55], but this also requires the discovery of an efficient marker for the question under investigation. A more in-depth exploration of the microbial diversity of the species (e.g. identification of associated species rather than bacterial morphotypes) may also be a successful alternative path to follow for Oscarellidae phylogeny and systematics [87].

## Supporting Information

Figure S1 **Different** ***in situ*** **color morphs of** ***Oscarella lobularis*** **and** ***O. tuberculata*** **species from the Marseille area.** A to D: *O. lobularis*. E to I: *O. tuberculata.*
(TIF)

Figure S2 **Evolution of some Oscarellidae characters, as optimized by Mesquite on a simplified consensus tree.** Double colored branches indicate non-determination of character-state in the branch. The squares below taxon names give character state in the considered taxon; no square means unknown (in this case, character-state in the corresponding branch is optimized according to character-states in related taxa). Clades A, B, C and D are indicated. (A) Characters: basement membrane, cinctoblastula larvae, multipolar ingression and asynchronous spermatogenesis. Presence in black; absence in white; (B) Character: Spherulous cells with paracrystalline inclusions. Presence in black; absence in white; (C) Characters: cortex, canal system and choanocyte chambers. Presence/leuconoid/diplodal in black; absence/sylleibid/eurypylous in white; (D) Character: vacuolar cells. Presence of two types in black; presence of one type in green; absence in white.
(EPS)

Figure S3 **Molecular diagnostic positions for** ***Oscarella lobularis*** **and** ***O. tuberculata*** **.** Partial alignments of mitochondrial markers (*atp6* and *tatC*) are provided and the diagnostic positions are identified by a black hexagon. A summary table for diagnostic positions for each marker for each species is also proposed.
(EPS)

Table S1 **PCR primers.** Names and sequences for primers used for rDNA and mitochondrial amplifications as well as references are provided.
(DOC)

Text S1 **Mesquite matrix for some morphological characters from** **Tables 2** **and** **3** **.** Characters and characters-states are detailed.
(PDF)

Text S2 **Mesquite matrix for V4 secondary structures for 18S rDNA.**
(PDF)
