# Supplementary figures and images for: Systematics and Molecular Phylogeny of the Family Oscarellidae (Homoscleromorpha) with Description of Two New Oscarella Species
Source: PLoS One. 2013 May 30;8(5):e63976. doi: 10.1371/journal.pone.0063976 (PMC3667853; doi:10.1371/journal.pone.0063976)

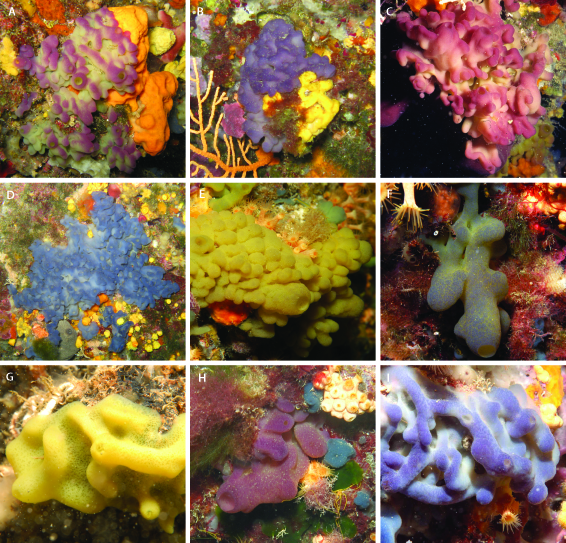

Supplement: Figure S1 — Different in situ color morphs of Oscarella lobularis and O. tuberculata species from the Marseille area. A to D: O. lobularis. E to I: O. tuberculata. (TIF) [file pone.0063976.s001.tif]
