# Supplementary material for: Systematics and Molecular Phylogeny of the Family Oscarellidae (Homoscleromorpha) with Description of Two New Oscarella Species
Source: PLoS One. 2013 May 30;8(5):e63976. doi: 10.1371/journal.pone.0063976 (PMC3667853; doi:10.1371/journal.pone.0063976)
Supplement: Table S1 — PCR primers. Names and sequences for primers used for rDNA and mitochondrial amplifications as well as references are provided. (DOC) [file pone.0063976.s004.doc]

| **Gene** | **Primer name** | **Sequence (5’-3’)** | **References** |
| --- | --- | --- | --- |
| **rDNA** |  |  |  |
| 18S | (F) S1 | AAC CTG GTT GAT CCT GCC A | [33] |
|  | (R) S2 | TGC AGG TTC ACC TAC AGA A | [33] |
|  | (F) D | ACT GTG AAA CTG CGA ATG GCT C | [6] |
|  | (R) G | CAC CTA CGG AAA CCT TGT TAC GCA | [6] |
|  | 18SHomo F | GYG AAA CTG CGA ATG GCY C | [6] |
|  | 18SHomo R | CTT GTT ACG ACT TTT ACY TCC TC | [6] |
| 28S | (F) X | GAA AAG AAC TTT GRA RAG AGA GT | [6] |
|  | (R) S2 | ATK CGY TTC CCT CCY AAC GG | [2] |
|  | (F) S1 | AGT CTT TCG CCC CTA TAC CCA | [6] |
|  | (R) Y | ACC CGC TGA ATT TAA GCA T | [2] |
|  | 28SHomo F1 | CAT ATC AAT AAG CGG AGG AA | [6] |
|  | 28SHomoF2 | GAG TCG GGT TGT TTG GGA | [6] |
|  | 28SHomoR | ATC GAT TTG CAC GTC AGA | [6] |
| **mtDNA** |  |  |  |
| *tatC* | Oscarella-rnl-f1 | CAA GAT AAG GAC GTT AAT AGG ATC | This study |
|  | Oscarella-trnQ-r1 | TTG TCG GGT CCA AGG TCC AAT GC | This study |
| *atp6* | Oscarella-atp6-f1 | TGA CAG CAG CAT ATT TTG ATC AG | This study |
|  | Oscarella-atp6-r1 | AAC ACA TAG GCC TGT ATT ACA GC | This study |
