# Supplementary material for: Systematics and Molecular Phylogeny of the Family Oscarellidae (Homoscleromorpha) with Description of Two New Oscarella Species
Source: PLoS One. 2013 May 30;8(5):e63976. doi: 10.1371/journal.pone.0063976 (PMC3667853; doi:10.1371/journal.pone.0063976)
Supplement: Text S1 — Mesquite matrix for some morphological characters from Tables 2 and 3 . Characters and characters-states are detailed. (PDF) [file pone.0063976.s005.pdf]

Mesquite version 2.74 (build 550)

======================

Character matrix editor for matrix "Character Matrix"

Type of matrix: Standard Categorical Data (compacted)

Number of characters: 12

Number of taxa: 16

Number of characters excluded: 0

Proportion of missing data: 0.02083333

Proportion of inapplicable codings: 0.0

	1	2	3	4	5	6	7	8	9	10	11	12
O. balibaloi	1	2	0	1	1	0	0	0	0	0	0	1
O. kamchatkensis	1	3	0	0	2	0	1	0	0	0	0	1
O. nicolae	1	1	1	0	1	0	3	0	0	0	0	1
P. jarrei	1	5	0	0	3	0	0	1	1	1	1	1
O. viridis	0	3	1	1	1	0	0	0	0	0	0	1
O. malakhovi	0	2	2	1	1	0	1	0	0	0	0	1
O. carmela	0	2	1	1	1	0	2	0	0	0	0	1
O. lobularis	0	3	0	2	0	0	0	0	0	0	0	1
O. tuberculata	0	2	1	1	0	0	0	0	0	0	0	1
O. rubra	0	2	0	1	1	0	4	0	0	0	0	1
O. sp2 pink	0	1	2	1	1	0	3	0	0	0	0	1
O. bergenensis	0	1	2	1	1	0	3	0	0	0	0	1
O. microlobata	1	4	0	1	1	1	0	0	0	0	1	1
O. sp. purple	0	2	1	1	1	0	0	0	0	0	0	1
C. candelabrum	0	8	?	?	?	0	0	1	1	1	1	1
P. simplex	0	6	?	1	1	1	0	1	1	1	1	1

Characters:

1 Spherulous cells with paracrystalline inclusions (0: absence; 1: presence)

2 bacteria (0: absence; 1: presence 1 type, 2: presence 2 types etc)

3 archaeocytes (0: absence; 1: presence)

4 vacuolar cells (0: absence; 1: presence 1 type, 2: presence 2 types etc)

5 granular cells (0: absence; 1: presence 1 type, 2: presence 2 types etc)

6 spherulous cells (0: absence; 1: presence)

7 locality (0: Mediterranean Sea, 1: N-W Pacific, 2: N-E Pacific, 3: E North Sea, 4: E Atlantic)

8 cortex (0: absence; 1: presence)

9 canal system (0: absence; 1: presence)

10 choanocytes chambers (0: absence; 1: presence)

11 density of bacteria (0: absence; 1: presence)

12 Basement membrane (0: absence; 1: presence)
