# Supplementary material for: Systematics and Molecular Phylogeny of the Family Oscarellidae (Homoscleromorpha) with Description of Two New Oscarella Species
Source: PLoS One. 2013 May 30;8(5):e63976. doi: 10.1371/journal.pone.0063976 (PMC3667853; doi:10.1371/journal.pone.0063976)
Supplement: Text S2 — Mesquite matrix for V4 secondary structures for 18S rDNA. (PDF) [file pone.0063976.s006.pdf]

Mesquite version 2.74 (build 550)

======================

Character matrix editor for matrix "Character Matrix2"

Type of matrix: Standard Categorical Data (compacted)

Number of characters: 7

Number of taxa: 25

Number of characters excluded: 0

Proportion of missing data: 0.0

Proportion of inapplicable codings: 0.0

	yellow	Orange	Light blue	Black	Grey	Dark blue	Red
O. tuberculata green	0	1	0	0	0	1	0
O. bergenensis	0	1	0	0	0	1	0
O. tuberculata purple	0	1	0	0	0	1	0
O. rubra	0	1	0	0	0	1	0
O. sp. purple	0	1	0	0	1	0	0
O. sp. pink	0	1	0	0	0	1	0
O. tuberculata blue	0	1	0	0	0	1	0
O. tuberculata pink	0	1	0	0	1	0	0
O. lobularis purple/ivory	0	0	0	0	0	1	0
O. lobularis pink	0	1	0	0	0	1	0
O. lobularis purple	0	1	0	0	0	1	0
O. lobularis blue	0	1	0	0	0	1	0
O. malakhovi	0	1	0	0	0	0	1
O. carmela	0	1	0	0	0	1	1
O. viridis	0	1	0	0	0	1	0
O. microlobata	0	1	1	0	0	0	0

O. balibaloi	0	1	1	1	0	0	0
O. nicolae	0	1	1	1	0	0	0
O. kamchatkensis	0	1	1	0	0	0	0
P. jarrei	0	1	0	0	0	0	0
Plakortis simplex	1	0	0	0	0	0	0
Plakortis halichondrioides	1	0	0	0	0	0	0
Corticium candelabrum	1	0	0	0	0	0	0
Corticium sp. palau	1	0	0	0	0	0	0
Corticium sp. vanuatu	1	0	0	0	0	0	0

0 : absence

1: presence
